# Systemic lupus erythematosus with antiphospholipid syndrome: Cardiovascular magnetic resonance for evaluation of cardiac hypertrophy

**DOI:** 10.31138/mjr.28.4.221

**Published:** 2017-12-22

**Authors:** Sophie I. Mavrogeni, Petros P. Sfikakis

**Affiliations:** 1 Onassis Cardiac Surgery Center, Athens, Greece,; 2 First Department of Propaedeutic and Internal medicine, Laikon Hospital, Athens University Medical School, Athens, Greece

**Keywords:** cardiovascular magnetic resonance, myocardial hypertrophy/fibrosis

This young, asymptomatic, female patient with a history of systemic lupus erythematosus (SLE) and antiphospholipid syndrome (APS) and normal cardiac enzymes was referred for CMR, due to hypertrophic cardiomyopathy, diagnosed by echocardiography. Indeed, the cine images identified the presence of hypertrophy, as it has been previously assessed by echocardiography (**Figure 1**), but the left ventricular ejection fraction was mildly reduced (LVEF=50%), which is unusual for early stages of hypertrophic cardiomyopathy, where LVEF is usually >70%. Furthermore, the late enhanced images (LGE), taken 15 min after the injection of gadolinium-DTPA, (**Figure 2**) identified the presence of subendocardial apical fibrosis (white area). This finding excludes the diagnosis of hypertrophic cardiomyopathy, characterised by fibrosis in the hypertrophic intraventricular septum and not by subendocardial fibrosis, which is typical of either macro- or microvascular coronary artery disease. The patient was catheterized and the coronary arteries were normal. Therefore, the lesion was attributed to microvascular disease, which is a common finding of cardiac involvement in APS patients. The coexisting hypertrophy was attributed to the reverse remodelling of LV, since the patient was not in any adequate cardiac medication until the CMR evaluation.

**Figure 1.**
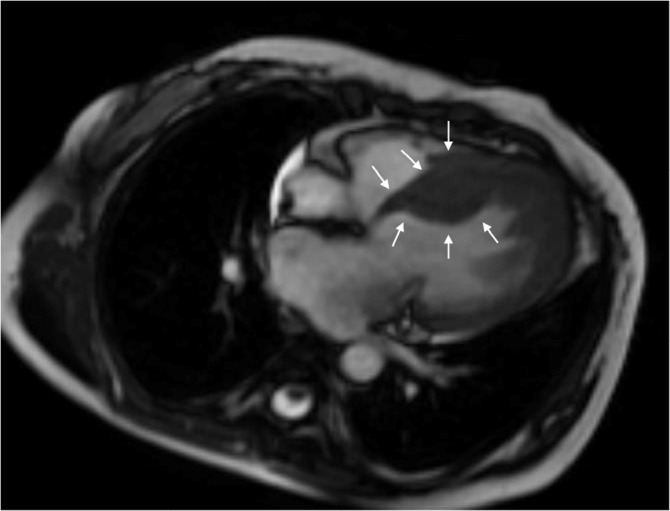
Four chamber cine image. The arrows identify the hypertrophied intraventricular septum.

**Figure 2.**
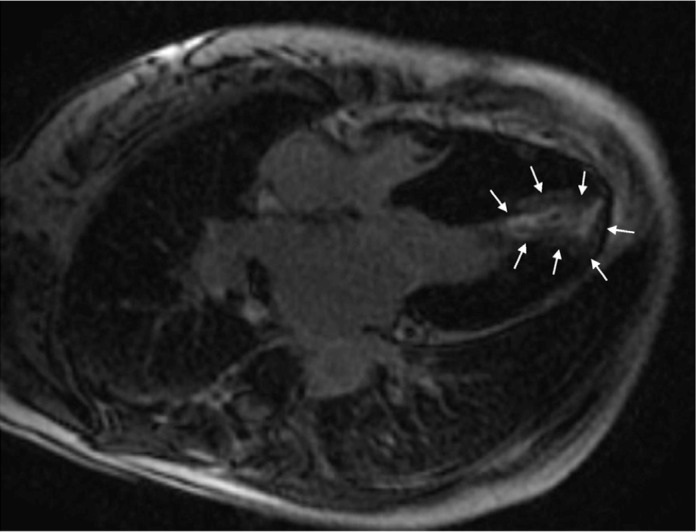
LGE image showing the apical subendocardial fibrosis (white area identified by arrows).

One may wonder why it is important to know the substrate behind cardiac hypertrophy: this knowledge will guide our final decision for patients’ risk stratification and further treatment. If the patient has hypertrophic cardiomyopathy and is asymptomatic, there is no need for coronary catheterization, and b-blocker is the right treatment. However, if the imaging background is compatible with coronary artery disease, coronary catheterization is needed and calcium blockers, ranolazine and ACE inhibitors should be included in the medication to avoid further ischemia, LV remodelling and LVEF reduction.

## DISCLAIMER

This research received no specific grant from any funding agency in the public, commercial, or not-for-profit sectors.

